# Multiple Spontaneous CSF Leaks in a Patient With Turner Syndrome: A Case Study

**DOI:** 10.1002/ccr3.72761

**Published:** 2026-06-01

**Authors:** Amelia L. Podolny, Catherine L. Kennedy, Meredith E. Adams, Andrew S. Venteicher

**Affiliations:** ^1^ University of Minnesota Medical School Minneapolis Minnesota USA; ^2^ Cooper Medical School at Rowan University Camden New Jersey USA; ^3^ Department of Otolaryngology—Head & Neck Surgery University of Michigan Ann Arbor Michigan USA; ^4^ Department of Neurosurgery University of Minnesota Minneapolis Minnesota USA

**Keywords:** CSF leak, encephalocele, intracranial hypertension, Turner Syndrome

## Abstract

Patients with Turner syndrome often have ear abnormalities which predispose them to chronic otitis media. They are also predisposed to develop intracranial hypertension. Both of these conditions can contribute to the development of skull base defects, which should be considered in patients with Turner syndrome presenting with them.

## 1 Introduction

Turner syndrome is characterized by partial or complete absence of one X chromosome and presents in approximately 1 in 2500 live births worldwide. Patients may have a short skull base; low‐set, cupped auricles with narrow external auditory canals; and eustachian tube dysfunction (ETD) [1]. These attributes contribute to conductive hearing loss and chronic otitis media that many adults with Turner syndrome experience [1]. However, there is little reported on the association between Turner syndrome and skull base disorders.

In this report, we describe the case of a 56‐year‐old woman with Turner syndrome with a history of chronic ear disease, who presented with cerebrospinal fluid (CSF) otorhinorrhea. She was found to have bilateral middle fossa encephaloceles and a right lateral sphenoid recess encephalocele which were managed by our lateral skull base surgical team. We present this case to highlight the importance of considering skull base disease in patients with Turner syndrome and history of chronic otitis media. This is the second case report describing a patient with Turner syndrome and history of chronic otitis media, and it is the first to link both of these conditions to idiopathic intracranial hypertension (IIH). This report describes a clinical case and the Institutional Review Board at the University of Minnesota assigned a determination of Not Human Research. No animals were used. Written informed consent was obtained from the patient regarding the publication of this case.

## 2 Case Presentation

A 56‐year‐old woman with Turner syndrome was referred for 8 months of otorhinorrhea. Previous karyotyping revealed her karyotype to be 46,X,i(Xq). On physical examination, she was found to be 134.6 cm tall, weighing 65.6 kg, with body mass index (BMI) of 36.19 kg/m^2^. She has a history of ETD and left cholesteatoma having undergone multiple prior surgeries in the early 2000s at an outside institution (right tympanoplasty and multiple left tympanomastoidectomies, including canal wall down mastoidectomy). Upon initial presentation, she reported 8 months of intermittent bilateral otorrhea and right clear rhinorrhea which persisted despite multiple courses of topical and oral antibiotics. On examination, she had sloughing wet debris in the right external auditory canal. Her tympanic membrane had been partially reconstructed with a cartilage graft, and the middle ear was filled with a clear, pulsing effusion without obvious perforation. The left ear mastoid cavity was filled with purulent debris which was removed, revealing a mature, skin‐covered meningoencephalocele which was slowly leaking CSF.

**FIGURE 1:**
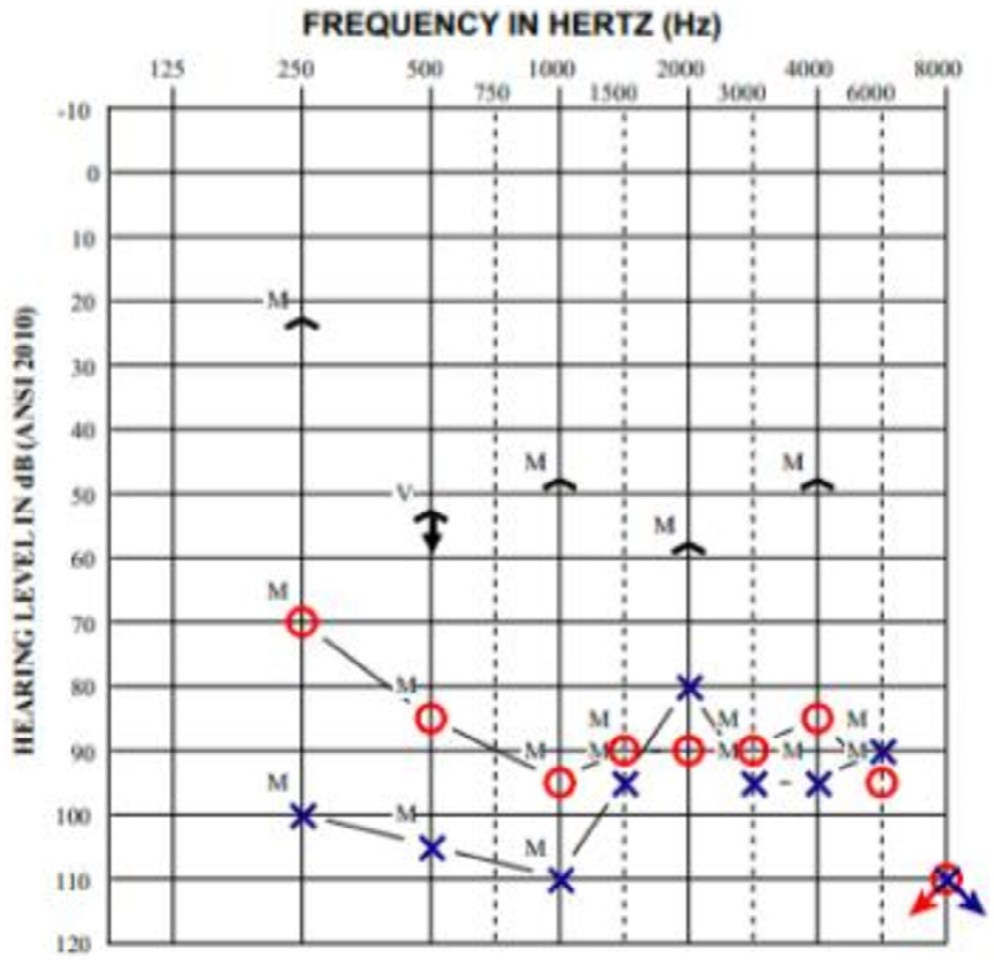
Audiogram of left ear (X) and right ear (O) from the patient's initial presentation, demonstrating right moderately severe sloping to profound unmeasurable hearing loss, and left profound rising to severe sloping to profound unmeasurable hearing loss.

**FIGURE 2:**
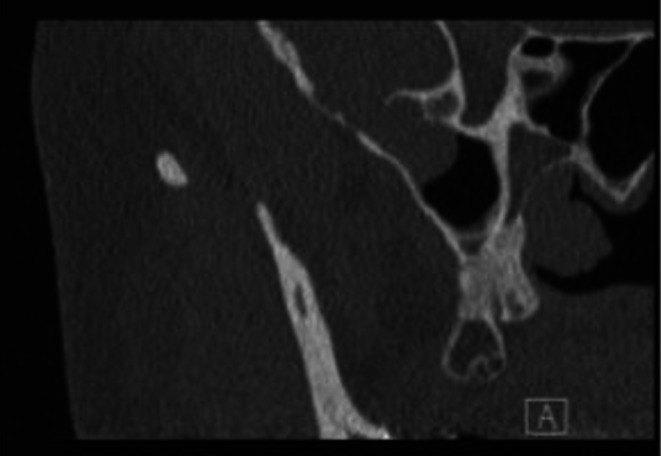
Right sphenoid sinus defect shown in coronal CT scan.

**FIGURE 3:**
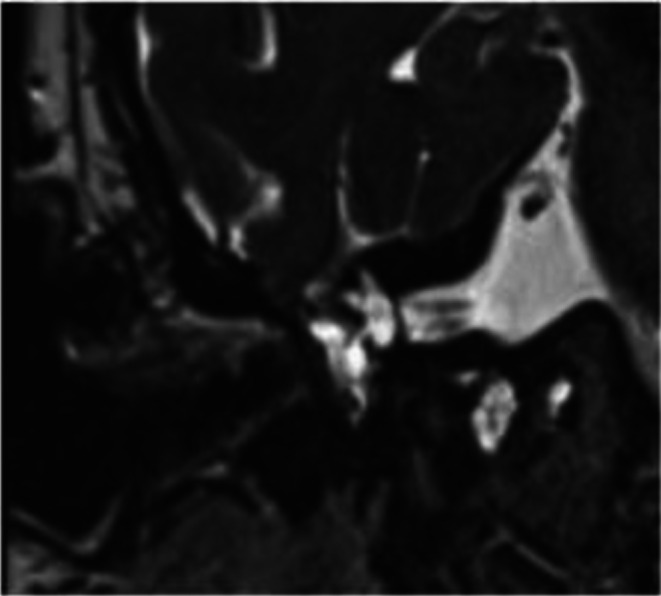
Right tegmen defect shown via coronal T2 MRI.

Her audiogram (Figure 1) demonstrated right moderately severe sloping to profound unmeasurable (likely mixed) hearing loss and left profound rising to severe sloping to profound unmeasurable (likely mixed) hearing loss. Word recognition was 52% on the right and 56% on the left. A computed tomography (CT) scan without contrast revealed bilateral tegmen defects with soft tissue densities protruding into the mastoids and sphenoid sinus wall defect (Figure 2). Magnetic resonance imaging (MRI) showed bilateral middle cranial fossa meningoencephaloceles and a meningoencephalocele protruding into the right sphenoid sinus (Figure 3).

**FIGURE 4:**
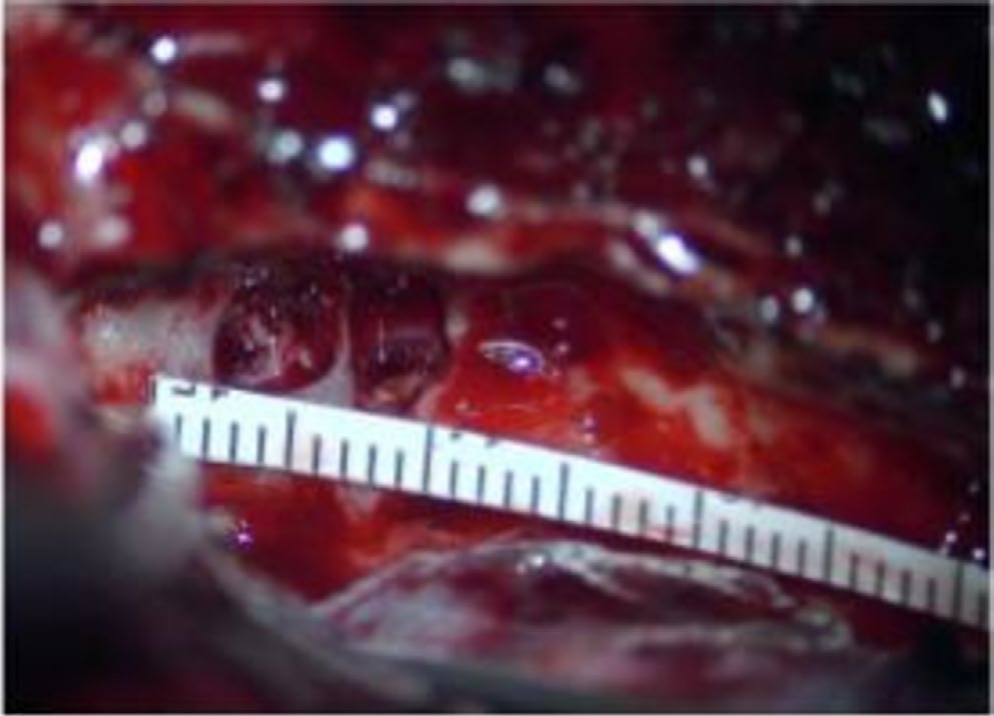
Measurement of two large encephaloceles located along the right lateral sphenoid recess.

**FIGURE 5:**
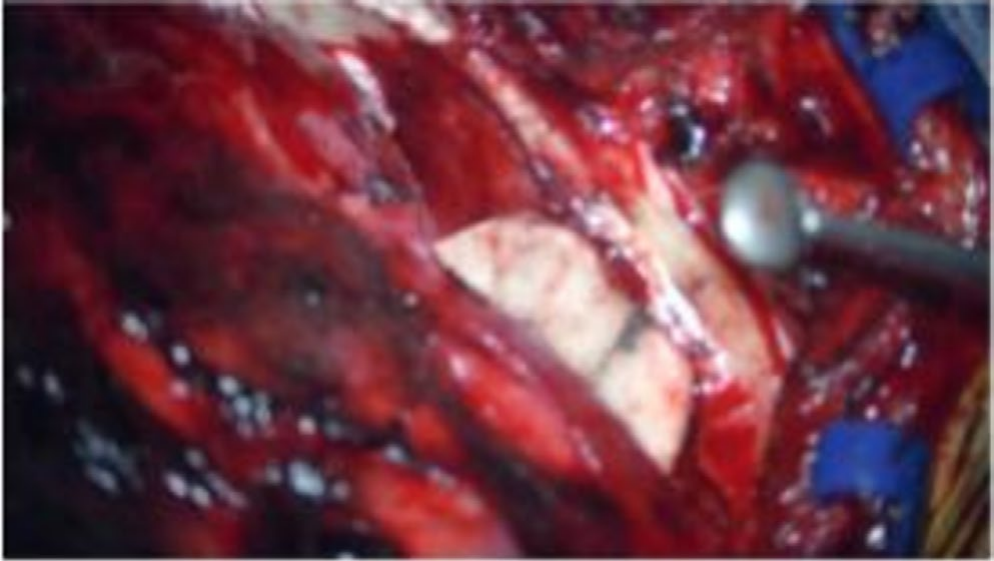
Placement of split thickness bone graft over the largest defect on the right middle fossa floor overlying the incus.

Our skull base surgery team proceeded with repair of the right anterior and lateral skull base defects, starting with a frontotemporal craniotomy extending from the pterion to the external auditory canal. The anterior skull base was first explored revealing two large encephaloceles and several small encephaloceles along the lateral sphenoid recess (Figure 4). The encephaloceles were cauterized and the dural openings were repaired. A portion of the encephalocele was left in situ and a pericranium graft was layered followed by a split thickness calvarial graft. Exploration of the right middle fossa floor revealed multiple small encephaloceles and a large encephalocele overlying the incus. The encephalocele was resected and the dural openings were repaired. A multilayer repair was performed with temporalis fascia, a split thickness calvarial bone graft, and duragen (Figure 5)—see Video 1 for details. Her postoperative course was uneventful and she was discharged on post‐op Day 3. She underwent subsequent lumbar puncture which revealed increased opening pressure of 22 cmH_2_O (normal < 20 cmH_2_O), suggesting a diagnosis of IIH.

**VIDEO 1:** Intraoperative video containing highlights from the surgical repair of the right anterior and lateral skull base defects. Video content can be viewed at https://onlinelibrary.wiley.com/doi/10.1002/ccr3.72761.

## 3 Discussion

Turner syndrome is a sex chromosome disorder that is associated with a narrow external auditory canal, shortened skull base, ETD, and soft palate dysfunction [1]. Given these craniofacial abnormalities, patients with Turner syndrome commonly experience middle ear pathology. In this report, a patient with Turner syndrome who has a history of chronic otitis media and multiple prior ear surgeries presented with several months of otorrhea which was attributed to otomastoiditis. It was only after referral to a quaternary medical center that her skull base defects were identified.

The presence of three encephaloceles is highly unusual, raising suspicion for undiagnosed IIH. IIH is linked to increased BMI (defined as BMI exceeding 25 kg/m^2^), sleep apnea, and chronic middle ear disease—all of which are seen more frequently in patients with Turner syndrome than the general population [2]. For reasons not fully understood, increased BMI is commonly seen in patients with Turner syndrome [3]. Bugjaska et al. have shown that although amino acid metabolism in patients with Turner syndrome differs from that of patients without chromosomal abnormalities, this metabolic difference does not explain the increased prevalence of high BMI seen in patients with Turner syndrome [3].

Sleep apnea is another risk factor for IIH which may contribute to the development of a spontaneous CSF leak [4]. Although a clear link between sleep apnea and Turner syndrome has not been established, patients with Turner syndrome tend to have multiple risk factors for developing sleep apnea (in addition to obesity as mentioned above). Compared to other patients, those with Turner syndrome have been found to have a shorter pharyngeal depth and brachycephaly, potentially increasing their susceptibility to developing sleep apnea [5].

The patient described in this report has multiple risk factors for encephalocele development which include obesity (patient's BMI = 36.19 kg/m^2^), IIH (patient's opening pressure = 22 cmH_2_O), chronic middle ear disease, and suspected sleep apnea. These risk factors stemming from her diagnosis of Turner syndrome provide a likely explanation for the development of her multiple encephaloceles. A high index of suspicion is needed to identify CSF otorrhea in patients with Turner syndrome given the high incidence of chronic middle ear disease, but otorrhea refractory to appropriate antimicrobial therapy should be investigated with imaging.

## 4 Conclusion

This report presents the case of a patient with Turner syndrome who developed multiple encephaloceles and underwent right frontotemporal craniotomy for approach to middle fossa repair of a lateral sphenoid recess encephalocele and middle fossa encephalocele. This patient exhibited multiple risk factors for IIH and encephalocele formation. In patients with risk factors for IIH who present with otorhinorrhea, skull base imaging with CT or MRI should be conducted to rule out encephalocele presence.

## Author Contributions

**Amelia L. Podolny:** investigation, visualization, writing – original draft, writing – review and editing. **Catherine L. Kennedy:** conceptualization, investigation, project administration, supervision, visualization, writing – review and editing. **Meredith E. Adams:** conceptualization, project administration. **Andrew S. Venteicher:** project administration, supervision, writing – review and editing.

## Funding

The authors have nothing to report.

## Ethics Statement

This case report was approved by the IRB of the University of Minnesota. Deidentified patient data may be made available upon request in accordance with privacy or ethical restrictions.

## Consent

Written informed consent from the patient was obtained regarding the publication of this case.

## Conflicts of Interest

The authors declare no conflicts of interest.

## Data Availability

The data that support the findings of this study are available on request from the corresponding author. The data are not publicly available due to privacy or ethical restrictions.
